# Growth reaction norms of domesticated, wild and hybrid Atlantic salmon families in response to differing social and physical environments

**DOI:** 10.1186/1471-2148-13-234

**Published:** 2013-10-28

**Authors:** Monica Favnebøe Solberg, Zhiwei Zhang, Frank Nilsen, Kevin Alan Glover

**Affiliations:** 1 Section of Population Genetics and Ecology, Institute of Marine Research, P.O. Box 1870, Nordnes, NO-5817 Bergen, Norway; 2 Department of Biology, University of Bergen, P.O. Box 7800 N-5020 Bergen, Norway; 3 Jiangsu Institute of Marine Fisheries, NanTong City, PR China

**Keywords:** Farmed escapees, Hybridization, Introgression, Common-garden, Reaction norm, Inter-strain competition, Heritability

## Abstract

**Background:**

Directional selection for growth has resulted in the 9-10th generation of domesticated Atlantic salmon *Salmo salar* L. outgrowing wild salmon by a ratio of approximately 3:1 when reared under standard hatchery conditions. In the wild however, growth of domesticated and wild salmon is more similar, and seems to differ at the most by a ratio of 1.25:1. Comparative studies of quantitative traits in farmed and wild salmon are often performed by the use of common-garden experiments where salmon of all origins are reared together to avoid origin-specific environmental differences. As social interaction may influence growth, the large observed difference in growth between wild and domesticated salmon in the hatchery may not be entirely genetically based, but inflated by inter-strain competition. This study had two primary aims: (i) investigate the effect of social interaction and inter-strain competition in common-garden experiments, by comparing the relative growth of farmed, hybrid and wild salmon when reared together and separately; (ii) investigate the competitive balance between wild and farmed salmon by comparing their norm of reaction for survival and growth along an environmental gradient ranging from standard hatchery conditions to a semi-natural environment with restricted feed.

**Results:**

The main results of this study, which are based upon the analysis of more than 6000 juvenile salmon, can be summarised as; (i) there was no difference in relative growth between wild and farmed salmon when reared together and separately; (ii) the relative difference in body weight at termination between wild and farmed salmon decreased as mortality increased along the environmental gradient approaching natural conditions.

**Conclusions:**

This study demonstrates that potential social interactions between wild and farmed salmon when reared communally are not likely to cause an overestimation of the genetic growth differences between them. Therefore, common-garden experiments represent a valid methodological approach to investigate genetic differences between wild and farmed salmon. As growth of surviving salmon of all origins became more similar as mortality increased along the environmental gradient approaching natural conditions, a hypothesis is presented suggesting that size-selective mortality is a possible factor reducing growth differences between these groups in the wild.

## Background

Hybridization between wild species and their domesticated or hatchery-reared conspecifics constitutes a potential threat to the genetic integrity of natural populations [1,2], and a thorough understanding of the consequences of introgression is crucial for successful conservation of these species. Wild/domesticated hybridization in Atlantic salmon *Salmo salar* L. is a current concern, due to the large numbers of salmon that escape from the domestic environment each year, and the very high proportions of escapees observed at the spawning grounds in some populations [3]. Although detection of escaped salmon in native populations is not synonymous with introgression [4,5], successful introgression has been documented in rivers in Ireland [6-9], Northeast America [10] and Norway [4,5,11]. In one of the most extensive of these studies [4], two rivers displayed highly significant temporal genetic changes when compared to expected genetic changes based upon simulations [12], which suggest strong introgression of farmed salmon. Recently, cumulative introgression of farmed salmon has also been quantified for the first time in wild populations [5]. Due to the fact that significant genetic differences have been observed between farmed and wild salmon for a range of traits [13-28], it is not surprising that there are significant concerns over the fitness related consequences in native populations where escapees have introgressed [29-32]. Therefore, comparative studies of quantitative traits along the wild/domesticated interface of this species can be used to gain a comprehensive understanding of the evolutionary principles of introgression and hybridization.

Somatic growth in salmonids is a highly polygenic trait [33], and has been the primary target of Atlantic salmon selection programs [13,34,35] since commercial production of this species was initiated in the late 1960’s [36]. Selection for increased growth has resulted in domesticated salmon outgrowing wild salmon when studied under standard hatchery conditions [13,15,16,20,28,37-40]. In the most recent growth study, which included the 9-10th generation of domesticated Atlantic salmon, farmed salmon outgrew wild salmon at the freshwater stage at a ratio of 2.9:1 [28]. However, in the wild, domesticated and wild salmon display more similar growth rates [41,42]. For example, the 8-9th generation of domesticated salmon, when studied in a natural river, outgrew wild salmon at the most by a ratio of 1.25:1 [41]. So why does farmed salmon outgrow wild salmon extensively in the hatchery, while not in the wild, and are the growth differences detected in the hatchery caused by additive genetic variation?

Social interaction and hierarchies are well documented in salmonids [43,44] where in general bigger and bold fish get better access to feed than smaller and shy fish. As individual differences in growth inflicted by social interaction do not solely reflect genetic differences in growth potential, this could lead to an overestimation of additive genetic variation between salmon of differing strains if they are reared in a communal environment. Comparative studies on the wild/domesticated interface are often performed by the use of common-garden experiments [16,28,45,46], where salmon of all origins are reared together and later assigned to origin by the use of DNA. This approach is often implemented to avoid origin-specific random environmental effects, as could be the case if salmon of differing origin were reared separately. However, as the common-garden design makes it hard to identify confounding effects of social interaction on the traits being studied, e.g., growth, it is valid to ask if the documented differences in growth between farmed and wild salmon are reflected by their additive genetic inheritance for growth or if inter-strain competition is influencing and potentially amplifying the observed growth differences. Would differences in growth between wild and farmed salmon be just as large if salmon of differing origins were reared separately and not communally? In order to answer this question, and to avoid overestimating genetic differences between salmon of wild and domesticated origin, the effect of social interaction and inter-strain competition upon growth in common-garden experiments should be clarified.

Growth rate is an essential factor for an optimal life history strategy in teleosts [47], and while the lack of natural selection in the domestic environment might allow extreme phenotypes to adapt to the predator-free environment, such phenotypes might be maladaptive in the wild and hence selected against, resulting in deviating optimal phenotypes between these environments. The domestic environment deviates from the natural environment in a multitude of ways, as high densities of salmon are reared in a predator-free environment with continuous access to feed. As a result, mortality in the domestic environment is low and mainly assigned to conditions at the site, rearing routines, and disease outbreaks [48]. In contrast, for salmonids in the natural freshwater environment mortality is high, 96.8 – 99.8% [14], as a result of natural selective forces, such as predation and competition [49-51]. In the wild, territorial and nutritional competition could be selecting against the slowest growing phenotypes, if small individuals are not gaining access to resources and as a consequence are more vulnerable to starvation, predation and parasites [51-55]. In addition, the risk of predation could be selecting against the fastest growing phenotypes, if they are associated with high-risk behaviour. In contrast, relaxed natural selection in the hatchery combined with directional selection for production related traits, has lead to reduced anti-predator responses in domesticated and hatchery reared salmonids [16,20,21,56] and increased aggressiveness [16,19,20]. Thus, it is possible that extreme phenotypes are selected against in the wild, reducing the large differences in relative growth rate in wild and farmed salmon that have been observed when they are both reared in the hatchery. Thus, elucidating the competitive balance between wild and domesticated salmon, under different environmental conditions, could benefit towards an understanding of why large difference in growth between wild and domesticated Atlantic salmon are detected in the hatchery environment, but not in the wild.

The present study had two primary aims; (i) investigate if the documented differences in growth between wild and farmed salmon are caused by genetic differences between the strains or influenced by social interaction due to the fact that they are communally reared; (ii) elucidate the competitive balance between wild, hybrid and farmed Atlantic salmon by comparing their norm of reaction for survival and growth along a gradient ranging from a standard hatchery environment to a semi-natural environment with restricted feed. In order to address these objectives, two separate experiments were conducted in 2011/2012. In experiment I, the effect of social interaction and inter-strain competition upon growth in a standard hatchery environment was investigated by comparing the growth of farmed, hybrid and wild salmon when reared together and separately from the eyed-egg stage. In experiment II, the effect of physical environment and nutritional competition on the expression of survival and growth of farmed, hybrid and wild salmon was investigated under standard hatchery conditions, hatchery conditions with restricted access to feed, and in a predator-free semi-natural environment. We predicted intra-strain competition to be as strong as inter-strain competition, and therefore to detect similar relative differences in body weight at termination between wild and domesticated salmon when reared separately and together. Further, we predicted that the relative difference in body weight at termination between surviving wild and domesticated salmon would decrease as the competition level increased along the environmental gradient, while approaching conditions similar to the natural environment.

## Methods

### Production of experimental families

The genetic material used in both experiments was produced in 2010. Wild Atlantic salmon from the river Figgjo (58°47′N, 5°38′E), and farmed salmon originating from the commercial Mowi strain were used to generate three experimental crosses; (i) ten pure wild families; (ii) ten pure farmed families; (iii) ten F_1_ hybrid families. Hybrid families were established by crossing farmed females with wild males. Thus the hybrid families were paternal and maternal half-siblings of the farmed and wild families, respectively. This hybrid design was chosen as it resembles the F_1_ hybrid design most likely to be observed in nature [42], and because the farmed salmon were larger and thus more eggs were available to produce both pure and hybrid families. The three experimental groups are from hereon referred to as farmed (Mowi), hybrid (Mowi x Figgjo) and wild (Figgjo).

The Figgjo river has the second largest wild salmon stock in Rogaland, Western Norway, with a female spawning population that exceeds the limit required to attain the rivers estimated carrying capacity [4,57]. The Mowi strain is the oldest Norwegian farm strain [36], established from large multi-sea winter fish collected from rivers in Hordaland and Sogn og Fjordane, river Vosso and river Årøy, respectively, as well as salmon caught at sea outside Western Norway [13,22]. The Mowi strain has been selected for increased growth, delayed maturation and fillet quality, in addition to other traits in more recent years [13]. Offspring of the approximately 10th generation were used as parents for the experiments here.

Wild salmon were caught by angling in the river Figgjo on October 15–17, 2010. These were immediately transferred to a local hatchery, and subsequently transported to Matre Research station on October 25, 2010. Unfertilized ova and milt from 10 female and 10 male farmed salmon (c. weight: 12–18 kg) were collected from the Mowi breeding station located at Askøy, and transported to the Matre Research Station. Wild salmon (females: *n* = 10, 2.24 ± 0.53 kg, mean ± S.D, males: *n* = 8, 1.98 ± 0.60 kg, mean ± S.D) were stripped upon arrival of the farmed gametes. All families were established on November 23, 2010, at the Matre Research Station (for family crosses see Additional file 1). From all parental fish adipose fin clips were collected for later parentage testing. Scale samples were also taken from the wild salmon and analysed to confirm that they were not escapees from farms [58].

**Figure 1:**
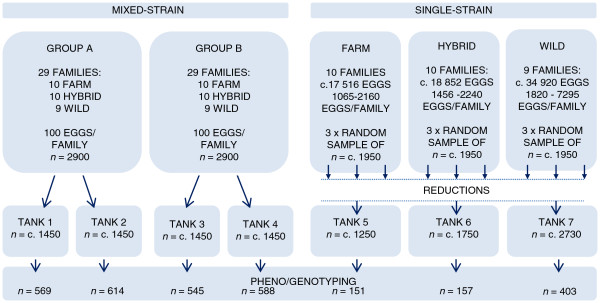
**Overview of the experimental design, experiment I.** The experimental period lasted for 44 weeks. Sampled individuals were sorted into smolts and non-smolts, based upon size and parr markings, and in the mixed-strain treatments and the single-strain treatment individuals were randomly sampled until 500 and 150 smolts, respectively, were sampled from each treatment tank. Out of the 3027 individuals sampled, 61 individuals were removed due to unsuccessful family assignment, growth malformations or sampling errors, leaving the total data set for growth comparisons consisting of 2966 individuals. The single-strain treatment initially consisted of three replicates per origin. However, to control for increasing biomasses these replicates were thinned as being merged into two replicates, where one replicate were later terminated due to rearing capacity.

**Figure 2:**
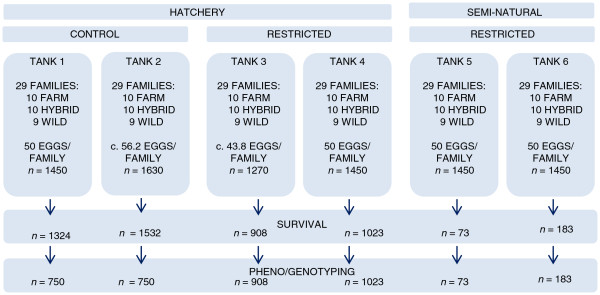
**Overview of the experimental design, experiment II.** The experimental period lasted for 20 weeks. The 750 individuals sampled from each of the hatchery control treatment replicates (tank 1 and 2) were randomly selected, while all surviving individuals were sampled from the restricted hatchery treatment (tank 3 and 4) and the restricted semi-natural treatment (tank 5 and 6). Out of the 5620 initial individuals in the restricted hatchery treatment and the restricted semi-natural treatment, 5568 were included as dead or alive in the survival analysis. Hence, 52 individuals (0.8 individual/family in tank 2 and 1 individual/family in tank 3) were excluded to control for the unbalanced design and the few surviving individuals that were not unambiguously assigned to a single family in the restricted hatchery treatment. Out of the 3687 individuals genotyped, 34 individuals were removed due to unsuccessful family assignment, growth malformations or sampling errors, leaving the total data set for growth comparison consisting of 2236 individuals.

All 30 families were incubated in single-family units until the eyed-egg stage. Dead eggs were picked daily and then shocked on January 31, 2011, to sort out dead eggs. One wild family was at this point excluded from the study, due to high egg mortality. Hence, the farmed, hybrid and wild origins were represented with 10:10:9 families respectively. Measurement of eggs from all families (diameter in mm) were taken on February 18, 2011. On February 22, 2011, fertilized eggs were sorted into the two experiments (Figures 1 and 2).

### Experiment I

In order to investigate the effect of social interaction and inter-strain competition between salmon of wild, hybrid and domesticated origin, salmon families were reared under standard hatchery conditions from the eyed-egg stage in February 2011, until March 2012. They were reared in; (i) mixed-strain tanks or (ii) single-strain tanks. Individual weight measurements upon termination of the experiment were collected in order to investigate the effect of inter-strain competition on growth. These treatments are from here on referred to as the mixed-strain treatment and the single-strain treatment respectively.

Experimental groups were transferred to 1 or 1.5 m^3^ tanks continuously supplied with fresh water on May 9, 2011 (for temperatures throughout the experimental period see Additional file 2). From May 10, 2011, fry were presented with commercial fish pellets 24 hours per day by automatic feeders. A standard feeding table for appropriate temperatures was used to calculate the feeding ration, and pellet sizes were adjusted to the mean fish weight, after weighing a sample of 100 individuals per tank. A combination of pellet sizes were used according to supplier’s protocol to ensure that all fish were given suitable feed. Experimental groups were from start-feeding onwards kept under 24 hours daily light, until being transferred onto a natural light regime in September 2011. For a schematic overview of the experiment, see Figure 1.

Each of the two replicate groups in the mixed-strain treatment (group A and B) consisted of 100 eggs per family (*n* = 2900/replicate). Each replicate was initially reared in 1.5 m^3^ tanks, but due to increasing biomass, each replicate was later transfer to 3 m^3^ tanks, before being divided in two equal portions (Figure 1). Thus, at the time of sampling, the mixed-strain treatment groups A and B each consisted of two replicates (total *n* = 4). In the single-strain treatment, eggs from all families of either farmed, hybrid or wild origin (10:10:9 families respectively) were reared together in mixed-family single-strain tanks (Figure 1). Approximately 1950 individuals per origin were randomly sampled in three 1 m^3^ tank replicates (*n* = 5850/strain). These replicates were transferred to three or more 1.5 m^3^ tanks to account for increasing biomass, and later merged into two 3 m^3^ tank replicates. Prior to termination of the experiment, one replicate per origin were terminated due to capacity. Thus, at the time of sampling the single-strain treatment consisted of one replicate per origin (Figure 1).

The experiment was terminated on March 14–16 and 19–20, 2012. Thus the experiment lasted for 44 weeks. Upon termination, individuals were sorted into smolts and non-smolts, based upon parr markings and body size. In the mixed-strain treatment, individuals were randomly sampled until 500 smolts were sampled from each of the four replicates. In the single-strain treatment, individuals were randomly sampled until 150 smolts were sampled from each of the three origins. Thus, sample size ranged from 545–614 individuals/tank in the mixed-strain treatment (48–99 individuals/family/treatment), and from 151–403 individuals/tank in the single-strain treatment (7–88 individuals/family/treatment). All sampled individuals were euthanized with an overdose of metacain (Finquel® Vet, ScanVacc, Årnes, Norway), wet weighed, fork length measured and caudal or adipose fin clipped. Fins were preserved in 95% ethanol, and all individuals were subsequently assigned to family using six DNA microsatellite markers.

### Experiment II

In order to elucidate the competitive balance between wild, hybrid and domesticated Atlantic salmon at the juvenile stage, salmon families of all origins were reared together from the eyed-egg stage in February 2011, until September 2011. They were reared under; (i) standard hatchery conditions; (ii) hatchery conditions with restricted access to feed and; (iii) in a semi-natural environment with restricted access to feed. Individual weight measurements were collected upon termination of the experiment and absolute or relative survival were recorded in order to examine the effect of nutritional competition between salmon of farmed, hybrid and wild origin in a predator-free environment. These treatments are from hereon referred to as the hatchery control treatment, the restricted hatchery treatment and the restricted semi-natural treatment respectively. For a schematic overview of the experiment see Figure 2.

Eyed eggs were planted in the semi-natural environment on March 10, 2011. The semi-natural environment consisted of a c. 31.4 m^2^ circular shaped passage (outer radius 3.5 m, inner radius 1.5 m), filled with gravel and hiding places. Eggs were planted c. 12 cm below the gravel, and water level and velocity were modified according to the levels documented at spawning areas of Atlantic salmon [59]. Eggs were planted in Vibert boxes (<500 eggs/box), approximately 3 m downstream of the water inlet of each tank, thus to ensure sufficient water flow. Automatic feeders were placed immediately beside the water inlet to ensure successful spreading of pellets in the circular tanks.

Rearing conditions of the experimental groups to be reared in the two hatchery treatments were similar to the rearing conditions of experiment I. Thus, start-feeding were initiated on May 10, 2011, while start-feeding in the semi-natural environment was initiated at the corresponding degree day (Table 1). All experimental groups were from start-feeding and throughout the experiment kept under 24 hours daily light. Each replicate initially consisted of 50 eggs per family (*n* = 1450/replicate). Upon start of the experimental treatment, approximately 180 individuals were accidentally transferred from a restricted hatchery treatment tank (tank 3, mean weight = 0.22 g/individual) to a hatchery control treatment tank (tank 2, mean weight = 0.22 g/individual). This was done in association with weighing fish to enable feeding ration computations. Thus, the four replicates in the hatchery environment were presented by 1450:1630:1270:1450 individuals respectively (Figure 2).

**Table 1:** Feeding regime, mortality and mean body weight during the experimental period, experiment II

	**Physical treatment**	**Tank**	***n *** **start**	**~week 0**			**Start-feeding (week 0 – 5)**				**Week 6 - 10**				**Week 11 - 15**				**Week 16 – 20 (termination)**					**M (%)**
				**M**	**W(g)**	**DD**	**F (%)**	**M**	**W(g)**	**DD**	**F (%)**	**M**	**W(g)**	**DD**	**F (%)**	**M**	**W(g)**	**DD**	**F (%)**	**M**	**W(g)**		**DD**	
																					**Mean**	**SD**		
Hatchery	1	1450	25	0.19	269	110	59	0.93	711	110	9	3.05	1128	110	14	9.61	1583	110	19	22.78	11.76	1996	8.7
control	2	1630	17	0.19	269	110	47	0.92	711	110	7	2.85	1128	110	9	10.06	1583	110	18	21.66	12.03	2017	6.0
Restricted	3	1270	18	0.19	269	75	58	0.91	711	25	25	1.48	1128	50	190	3.58	1583	50	71	7.76	6.3	2006	28.5
hatchery	4	1450	28	0.19	269	75	37	0.94	711	25	57	1.48	1128	50	239	2.71	1583	50	66	7.07	6.11	2028	29.4
			**Week 1**			**Start-feeding (week 1 – 8)**				**Week 9 - 12**				**Week 13 - 18**				**Week 19 – 20 (termination)**					
			**M**	**W(g)**	**DD**	**F (%)**	**M**	**W(g)**	**DD**	**F (%)**	**M**	**W(g)**	**DD**	**F (%)**	**M**	**W(g)**	**DD**	**F (%)**	**M**	**W(g)**		**DD**	
																				**Mean**	**SD**		
Restricted	5	1450	NA	NA	269	75	NA	NA	712	25	NA	NA	1125	25	NA	NA	1602	25	1377	11.01	5.98	1795	95.0
semi-natural	6	1450	NA	NA	269	75	NA	NA	712	25	NA	NA	1125	25	NA	NA	1602	25	1267	9.08	4.51	1795	87.4

M; mortality (*n*). W; weight (gram). DD; degree day post median time of hatch. F; feeding regime (percent of the recommended feeding regime given by the commercial industry); M (%); percentage mortality throughout the experiment. Feed were adjusted once a week in tank 1 – 4, while tank 5 and 6 were adjusted at the corresponding degree day. Due to high mortality in the restricted hatchery treatment, the feeding regime was revised in week 11, while kept constant in the restricted semi-natural treatment. Weight measurements during the experimental period is based upon a bulk weight of 100 individuals, while weight at termination is based upon the sampled individuals that were identified to family for further growth comparisons. Initially each tank contained 1450 individuals, while in week 1, approximately 180 individuals were accidentally transferred from tank 3 to tank 2 during weight measurement. In week 1, mean individual weight in tank 1 – 4 was similar (i.e., 0.22 gram). Weight measurements are given for week 5, 10, 15 and 20, respectively.

The hatchery control treatment (*n* = 2) were reared according to standard hatchery conditions, feed *ad libitum* by providing a feed ration of 110% of the recommended ration (Table 1). Salmon in the restricted hatchery treatment (*n* = 2) and the restricted semi-natural treatment (*n* = 2) were given a reduced feed ration, 75% of the recommended ration during start feeding, then initially 25% of the recommended ration throughout the remaining experimental period (Table 1). Rations in the hatchery environment were adjusted once a week, while ration in the semi-natural environment was adjusted at the corresponding degree day. Mortality was recorded daily in the hatchery environment, although dead individuals were not assigned to family. Mean fish weight and mortality rates in the restricted hatchery treatment were used as an estimate for feed measurements in the restricted semi-natural treatment. Due to high mortality in the restricted hatchery treatment, feed ration was from week 11 and until termination increased to 50% of the recommended ration, while ration in the restricted semi-natural treatment was kept constant at 25% of the recommended ration throughout the experimental period (Table 1).

The experiment was terminated on September 26–30, 2011. Thus the experiment lasted for 20 weeks. In the hatchery control treatment, where mortality was low, 750 individuals were randomly sampled from each of the replicates (Figure 2). All surviving individuals were sampled in the restricted hatchery treatment (*n* = 908 and 1023), as well as in the restricted semi-natural treatment (*n* = 73 and 183) (Figure 2). Thus, growth was investigated based upon weight measurement of a representative sample for the control treatment in general, while in the restricted hatchery treatment and the restricted semi-natural treatment only growth of surviving individuals was investigated. All individuals were sampled in the same manner as in experiment I.

### Animal ethics

The experiments were performed in accordance with the general guidelines for animal studies, the Animal Research Reporting In Vivo Experiments (ARRIVE) guidelines [60]. The experimental protocols (permit number 3451 and 4268) were approved by the Norwegian Animal Research Authority (NARA). Welfare and use of experimental animals was performed in strict accordance with the Norwegian Animal Welfare Act of 19th of June 2009, in forced on the 1st of January 2010, while all personnel involved in the experiment had undergone mandatory training approved by the Norwegian Food Safety Authority.

### Genotyping and parentage testing

DNA from a total of 6727 individuals was extracted in 96 well plates using a Qiagen DNeasy®96 Blood & Tissue Kit, following procedures recommended by the manufacturer. Parental DNA was extracted twice, to ensure correct genotyping. Two randomly assigned blank wells were included on each 96-well plate to ensure a unique identification of the plate. Six microsatellite loci were amplified in one multiplex PCR; *SsaF43* [GenBank:U37494] [61], *Ssa197* [GenBank:U43694.1] [62], *SSsp3016* [GenBank:AY372820], *MHCI*[63], *MHCII*[64] and *SsOSL85* [GenBank:Z48596.1] [65]. PCR products were analysed on a ABI Applied Biosystems ABI 3730 Genetic Analyser. Genotypes were identified using GeneMapper V4.0., with manual control of scored alleles. All offspring were assigned to family by the use of FAP Family Analysis Program v3.6 [66]. This program has been used on several occasions for parentage testing common-garden studies using these facilities [28,67,68]. These genetic markers have revealed very low genotyping errors in this laboratory [69] and are routinely used in association with a genotyping service for the Norwegian legal authorities to identify the farm of origin for escapees [70,71]. In order to validate genotyping quality, 77 individuals were randomly selected for re-genotyping, where all gave identical genotype and parentage assignment on the second analysis.

### Statistical analyses

All statistical analyses were performed using R version 2.15.3 [72], with critical P-values set to 0.05, unless otherwise stated.

#### Experiment I – growth

In order to investigate the influence of social interaction and inter-strain competition upon growth in salmon of farmed, hybrid and wild origin, a linear mixed effects (LME) model were fitted using the *lmer* function in the lme4 package [73]. We first tested for effects of social treatment (S), fish origin (O) and egg size (E) upon body weight at termination (*Y*), with tank (t) as a random intercept factor nested within group (G) and/or social treatment, while allowing the intercept of families (f) to randomly vary across treatments, i.e., random slope (_f_S). All interaction terms between the fixed effects were included in the full model;

$$\begin{array}{l} Y=\alpha +\beta_{1}S+\beta_{2}O+\beta_{3}E+\beta_{4}\mathrm{SO}+\beta_{5}\mathrm{SE}+\beta_{6}\mathrm{OE}+\beta_{7}\mathrm{SOE}\\ \;+b_{t}{}_{\left(\left(G\right)S\right)}+b_{f}+b_{f}S+\epsilon \;\left(1\;\mathrm{Full}\right) \end{array}$$

where *ϵ* is a random error. The response variable, i.e., body weight at termination, was log-transformed (log_10_), as the difference in weight between treatment *y* (e.g., mixed-strain treatment) and *x* (e.g., single-strain treatment) of value *z* will equal a greater portion of the weight in treatment *y* if the value of *y* is small than if it is large [74-76]. A constant was added so that all values of the response variable were above 1 prior to the transformation.

Model selection were performed by first including fixed effects of the full model in a linear model, then random effect structures were added and the best structure identified based upon Akaike Information Criterion (AIC) values, while using Restricted Maximum Likelihood (REML) estimators [77]. Once the random effect structure were identified, the fixed effect structure were fitted by backward selection based upon AIC values, while using Maximum Likelihood (ML) estimators [77]. Models displaying AIC values of ± 2 were considered equally good and by the principle of parsimony, the simplest model that performed best were selected. Thus, insignificant variables were removed from the model, interaction terms before the variables themselves, until no further improvement of the model fit were detected;

$$Y=\alpha +\beta_{1}S+\beta_{2}O+\beta_{3}\mathrm{SO}+b_{\;t}{}_{\left(\left(G\right)S\right)}+b_{f}+\epsilon \;\left(1\;\mathrm{Selected}\right)$$

For AIC comparisons of the LME model, see Additional file 3. For parameter estimates of the selected model fitted using REML estimation, i.e., t-statistics retrieved from the summary output of the LME, see Additional file 4. P-values of the fixed effects were calculated from the F-statistics of the selected LME model, fitted using ML estimation. The F-value and the numerator degrees of freedom (k-1, where k is the number of factor levels) were retrieved from the anova output of the LME model. Denominator degrees of freedom were calculated as N- k, where N, conservatively, was set to the smallest sample size detected in any of the three origins in any of the two treatments. Differences in performance between salmon of specific origins were estimated by re-running the selected model while excluding one of the three genetic origins at a time (see Additional file 5). Multiple comparisons were corrected for by the Bonferroni correction, giving an adjusted significance level of 0.017 (α = P/3).

In order to investigate stability of family performance across social treatments, Pearson correlations were conducted between mean family log_10_-weight in the mixed-strain treatment and the single-strain treatment. Pearson correlation tests were performed separately for each origin, and the single-strain replicate were compared to each of the four mixed-strain replicates.

#### Experiment II – growth

In order to investigate the influence of physical environment and nutritional competition upon growth of surviving farmed, hybrid and wild salmon, linear mixed effects (LME) models were fitted pair wise for the three physical treatments constituting the environmental gradient, i.e., three models in total. We first tested for effects of physical treatment (P), fish origin (O) and egg size (E) upon body weight at termination (*Y*), with tank (*t*) as a random intercept factor nested within physical treatment, while allowing the intercept of families (f) to randomly vary across treatments, i.e., random slope (_f_P). All interaction terms between the fixed effects were included in the full model;

$$\begin{array}{l} Y=\alpha +\beta_{1}P+\beta_{2}O+\beta_{3}E+\beta_{4}\mathrm{PO}+\beta_{5}\mathrm{PE}+\beta_{6}\mathrm{OE}+\beta_{7}\mathrm{POE}\\ \;+b_{t}{}_{\left(P\right)}+b_{f}+b_{f}P+\epsilon \;\left(2.1‒2.3\;\mathrm{Full}\right) \end{array}$$

where *ϵ* is a random error. The response variable, i.e., body weight at termination, was log-transformed (log_10_), while a constant were added prior to the transformation. Model selection was performed as described above by removing insignificant variables until no further improvement of the model fits were detected;

$$Y=\alpha +\beta_{1}P+\beta_{2}O+\beta_{3}\mathrm{PO}+b_{\;t}{}_{\left(P\right)}+b_{f}+b_{f}P+\epsilon \;\left(2.1\;\mathrm{Selected}\right)$$

when fitted for salmon in the hatchery control treatment and the restricted hatchery treatment;

$$Y=\alpha +\beta_{1}P+\beta_{2}O+\beta_{3}\mathrm{PO}+b_{\;t}{}_{\left(P\right)}+b_{f}+\epsilon \;\left(2.2\;\mathrm{Selected}\right)$$

when fitted for salmon in the hatchery control treatment and the restricted semi-natural treatment;

$$Y=\alpha +\beta_{1}P+\beta_{2}O+b_{\;t}{}_{\left(P\right)}+b_{f}+b_{f}P+\epsilon \;\left(2.3\;\mathrm{Selected}\right)$$

when fitted for salmon in the restricted hatchery treatment and the restricted semi-natural treatment.

For AIC comparisons of the LME models and parameter estimates of the selected models, see Additional files 3 and 4. Calculation of P-values of the fixed effects and investigation of performance of salmon of specific origins (see Additional file 5) were performed as described above.

In order to investigate stability in family performance across the physical hatchery environments, Pearson correlations were performed between mean family log_10_-weight in the hatchery control treatment and the restricted hatchery treatment. Again, Pearson correlation tests were performed separately for each origin, and both replicates in the hatchery control treatment were compared to the two replicates in the restricted hatchery treatment. Pearson correlation tests were not performed across the restricted semi-natural treatment, due to the small sample size of some families.

#### Experiment II – sampling and mortality

In order to test for differences in observed mortality between the replicates in the hatchery control treatment, as well as to test for differences in sampling frequency of farmed, hybrid and wild salmon within the hatchery control subsamples, chi-square (*x*^*2*^) tests for given probabilities, based upon numbers, was performed.

In order to investigate the influence of different effects upon survival in the restricted hatchery treatment and the restricted semi-natural treatment where exact mortality was known, a generalized linear mixed effect model (GLMM) was fitted using the *lmer* function in the lme4 package [73]. We first tested for effects of physical treatment (P), origin (O) and egg size (E) upon survival (Y), with tank (t) as a random intercept factor nested within physical treatment, while allowing the intercept of families (f) to randomly vary across treatments, i.e., random slope (_f_P). All interaction terms between the fixed effects were included in the full model:

$$\begin{array}{l} \mathrm{logit}\;\left(Y\right)=\alpha +\beta_{1}P+\beta_{2}O+\beta_{3}E+\beta_{4}\mathrm{PO}+\beta_{5}\mathrm{PE}+\beta_{6}\mathrm{OE}\\ \;+\beta_{7}\mathrm{POE}+b_{t}{}_{\left(P\right)}+b_{f}+b_{f}P+\epsilon \;\left(3\;\mathrm{Full}\right) \end{array}$$

where *ϵ* is a random error. Due to survival being binary data, the binomial distribution was selected with a logit link function, and models were fitted using the Laplace approximation. Identification of the random effect structure was done by performing likelihood ratio tests (LRT) on the full fixed effects model. Then the fixed effects structure were identified by backward model selection, based upon AIC values [78]. Thus, insignificant variables were removed from the model, interaction terms before the variables themselves, until no further improvement of the model fit were detected:

$$\begin{array}{l} \mathrm{logit}\;\left(Y\right)=\alpha +\beta_{1}P+\beta_{2}O+\beta_{3}E+b_{t}{}_{\left(P\right)}+b_{f}\\ \;+b_{f}P+\epsilon \;\left(3\;\mathrm{Selected}\right) \end{array}$$

For model selection of the GLMM, see Additional file 6. Parameter estimates from the selected model were obtained by performing a Wald *Z*–test. Differences in probability of survival between salmon of specific origins were estimated by re-running the final model while excluding one of the three genetic origins at a time (see Additional file 7). Multiple comparisons were corrected for by the Bonferroni correction, giving an adjusted significance level of 0.017. To control for the unbalanced design and the few surviving individuals that were not unambiguously assigned to a single family in the restricted hatchery treatment, the estimated sample size per family was set to 43 individuals in tank 3 and 49 individuals in tank 4 (see Figure 2).

### Heritability of body weight (experiment I and II)

In order to investigate the portion of phenotypic variance attributed to genetic variation in salmon of farmed, hybrid and wild origin, heritability *h*^*2*^ of body weight (log_10_) was calculated as;

$$h^{2}=V_{A}/V_{P},$$

where V_A_ is the additive genetic variance and V_P_ is the phenotypic variance. Heritability estimates was calculated in the mixed-strain treatment of experiment I and in the hatchery control treatment and the restricted hatchery treatment of experiment II. To control for half-sibling families within the genetic origins, variance components were estimated from the pedigree of the data by fitting a generalized linear mixed model using Markov chain Monte Carlo methods (MCMCglmm) from the MCMCglmm package [79], i.e., the animal model [80,81]. One model was fitted per origin, per treatment, i.e., nine models in total. The full model included the fixed effect of egg size (E) upon body weight (log_10_) at termination (Y), with tank (t) and *Animal* (*a*) as random intercept effects:

$$Y=\alpha +\beta_{1}E+b_{t}+b_{a}+\epsilon \;\left(4.1‒4.9\;\mathrm{Full}\right)$$

where *ϵ* is a random error. *Animal* (*a*) is the additive genetic merit of an individual, i.e., the breeding value [80,81]. Thus, V_A_ is the estimated variance in breeding values [80]. The fixed effect egg size was not considered significant in any of the model as its posterior distribution overlapped zero [80], while model selection on the random effect structure, by the use of the Deviance Information Criterion (DIC), revealed significant tank effects in all experimental groups (for DIC comparisons see Additional file 8):

$$Y=\alpha +b_{t}+b_{a}+\epsilon \;\left(4.1‒4.9\;\mathrm{Selected}\right)$$

Weakly informative priors for the animal model were generated by equally partitioning phenotypic variance (V_P_) into the genetic and residual components, while placing little weight on the values specified by the priors, i.e., with a low degree of belief [80]. Priors with stronger degree of belief and with different partitioning of the phenotypic variance between the components were also tested. We settled on the weakly informative priors yielding conservative heritability estimates. Each model was run for 8,000,000 iterations with the first 300,000 iterations excluded as burn-in, and was thereafter sampled every 7000 iterations. Convergence of the model was checked by calculating autocorrelations among the samples of the posterior distributions [80]. As a measure of precision of the heritability estimates, credibility intervals were calculated as 95% highest posterior density (HPD) intervals using the *HPDinterval* function in the lme4 package [73].

## Results

### Experiment I – the effect of inter-strain competition upon growth

#### Genotyping and parentage testing

Out of the 3027 individuals sampled, 25 individuals were not selected for parental assignment due to a documented sampling error. A further 13 individuals were not assigned to family, either due to overlapping composite genotypes between family pairs, or due to genotyping errors. Thus, a total of 2989 individuals were unambiguously assigned to family. After parental assignment, 23 individuals were identified as outliers and post hoc excluded from the data set. These individuals displayed growth malformations and/or extreme condition factor values. The final data set consisted of 2966 individuals.

#### Growth in mixed-strain and single-strain tanks

**Figure 3:**
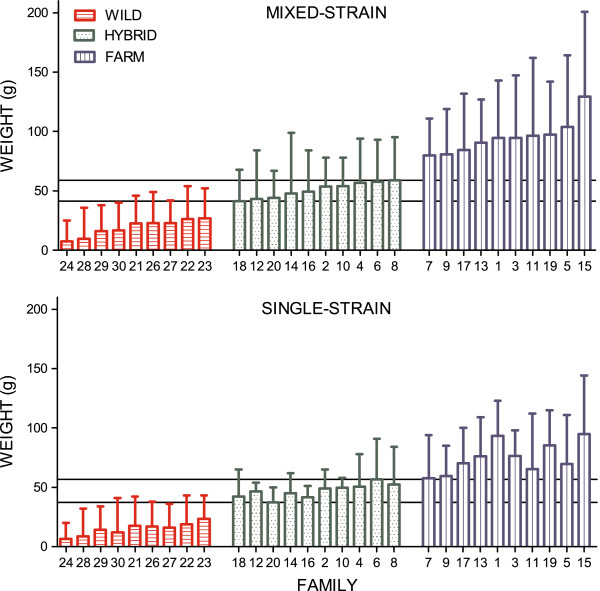
**Mean family weight of salmon of all origin reared separately and together, experiment I.** Mean family weight (g) of the farmed, hybrid and wild families reared in the mixed-strain treatment and the single-strain treatment. Replicated tanks in the mixed-strain treatment are pooled. Growth was significantly lower in the single-strain treatment; however relative weight between wild and farmed salmon were similar in both treatments. There is no overlap in mean family weight of the wild, hybrid and farmed families in any of the treatments. Families are ranked by their mean family weight in the pooled mixed-strain treatment, by increasing order. Lines represent the mean of the smallest and largest hybrid family within each treatment. Error bars show the range. See Additional file 9 for mean family weight in all four mixed-strain replicates.

In general, body weight of all salmon was significantly higher in the mixed-strain treatment than in the single-strain treatment (Tables 2 and 3; Figure 3). Farmed salmon were significantly larger than wild salmon, and hybrids displayed intermediate growth that was significantly different to both the farmed and wild salmon (Tables 2 and 3; Figure 3).

**Table 2:** **Weight measurements of *Salmo salar *** **L. of farmed, hybrid and wild origin, experiment I**

**Social treatment**	**Origin**	**Group**	**Tank**	***n***	**W (g)**			**L (cm)**			**K**		
					**Mean**	**Median**	**SD**	**Mean**	**Median**	**SD**	**Mean**	**Median**	**SD**
Mixed-strain	Farm	A	1	211	93.84	91	23.6	19.44	19.4	1.75	1.25	1.2	0.07
		A	2	170	94.22	92	26.2	19.49	19.5	1.85	1.24	1.2	0.07
		B	3	173	96.84	94	23.77	19.74	19.7	1.58	1.24	1.2	0.08
		B	4	149	96.16	95	23.16	19.58	19.5	1.71	1.26	1.3	0.07
	Hybrid	A	1	211	50.73	52	15.46	15.75	16.1	1.79	1.25	1.2	0.07
		A	2	208	48.99	49	13.67	15.7	15.9	1.7	1.22	1.2	0.07
		B	3	205	52.54	54	15.66	16.12	16.6	1.89	1.21	1.2	0.1
		B	4	208	51.25	51.5	15.76	15.91	16.2	1.93	1.23	1.2	0.1
	Wild	A	1	144	18.61	14.5	11.61	11.13	10.45	2.24	1.16	1.2	0.13
		A	2	233	18.65	14	11.72	11.17	10.5	2.42	1.16	1.2	0.13
		B	3	125	21.94	24	12.13	11.82	12.4	2.41	1.16	1.2	0.11
		B	4	222	19.18	17	11.5	11.2	10.8	2.24	1.19	1.2	0.13
Single-strain	Farm	NA	5	150	74.99	72	23.12	17.98	18.05	1.93	1.24	1.2	0.07
	Hybrid	NA	6	154	47.35	47	12.62	15.57	15.65	1.5	1.22	1.2	0.1
	Wild	NA	7	403	14.56	10	10.04	10.44	9.7	2.23	1.08	1.1	0.13

Condition factor (K), length (cm) and weight (g), with standard deviations.

**Table 3:** **Summary of the LME models testing for differences in weight(log**_**10**_**) at termination, experiment I and II**

**Experiment**	**Model**	**Social or physical treatment**	**Fixed effect**	**DFn**	**DFd**	**Sum Sq**	**F**	**P**
I	1	Mixed-strain vs. single-strain	Social treatment	1	148	0.9	28.3	<0.0001
			Origin	2	147	7.4	114.7	<0.0001
			Social treatment x origin^1^	2	147	0.3	4.8	0.017
II	2.1	Hatchery control vs. restricted hatchery	Physical treatment	1	425	6.1	157.9	<0.0001
			Origin	2	424	9.1	117.7	<0.0001
			Physical treatment x origin^2^	2	424	0.9	11.5	<0.0001
	2.2	Hatchery control vs. restricted semi-natural	Physical treatment	1	35	2.0	99.2	<0.0001
			Origin	2	34	4.7	118.0	<0.0001
			Physical treatment x origin	2	34	1.3	32.3	<0.0001
	2.3	Restricted hatchery vs. restricted semi-natural	Physical treatment	1	35	0.6	11.0	0.0009
			Origin	2	34	4.6	43.7	<0.0001

F-statistics of the selected linear mixed effects models, fitted using ML estimation. DFn; numerator degrees of freedom. DFd; denominator degrees of freedom. Differences in body weight at termination between salmon of farmed, hybrid or wild origin were investigated by re-running the selected models while excluding one of the three genetic origins at a time, while multiple comparisons were corrected for by the Bonferroni correction, giving an adjusted significance level of 0.017. ^1^ The interaction treatment x origin were not significant in the farmed and wild salmon (DFn = 1, DFd = 148, Sum Sq = 0.0006, F = 0.015, P = 1). ^2^ The interaction treatment x origin were not significant in the hybrid and farmed salmon (DFn = 1, DFd = 425, Sum Sq = 0.0014, F = 0.036, P = 1).

**Figure 4:**
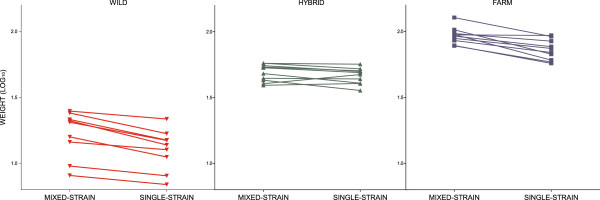
**Growth reaction norms between the mixed-strain treatment and single-strain treatment, experiment I.** Family weight (log_10_) norm of reaction between the two rearing treatments, for salmon of farmed, hybrid and wild salmon. Replicated tanks are pooled. Fish sizes were significantly higher in the mixed-strain treatment than in the single-strain treatment, and the elevation of the reaction norms were significantly different between salmon of farmed, hybrid and wild origin. The slopes between treatments were similar in the wild and farmed salmon, while the flatter slopes displayed by the hybrid salmon were significantly different to the slopes displayed by both the farmed and wild salmon. A significant correlation between mean family weight in the mixed-strain treatment and mean family weight in the single-strains treatment were detected in salmon of all origin.

In general, farmed and wild salmon displayed similar growth reaction norm slopes between the mixed-strain treatment and the single-strain treatment, only differing in elevation (Table 3; Figure 4; Additional file 5). Thus, the relative difference in weight between wild and farmed salmon were similar both when reared together and when reared separately. In general, the slopes displayed by the hybrid salmon were significantly flatter than the slopes displayed by both the farmed and wild salmon (Table 3; Figure 4; Additional file 5). Heterogeneity of variance among tanks (see Additional file 9) and families was detected and controlled for in the final LME model, while families did not differ in their variance across the two rearing treatments (Additional file 3). Egg size did not have a significant effect upon body weight at termination, thus the inclusion of egg size in the final LME models did not improve the fit (Additional file 3).

No overlap in mean family weight between the wild, hybrid and farmed salmon families were detected in the mixed-strain treatment, nor in the single-strain treatment (Figure 3). A significant correlation between mean family weight in the mixed-strain treatment and mean family weight in the single-strain treatment were detect in salmon of all origins (Wild: *n* = 36, Pearson r = 0.91, P <0.0001; Hybrid: *n* = 40, Pearson r = 0.67, P <0.0001; Farm: *n* = 40, Pearson r = 0. 61, P <0.0001; Figure 4).

### Experiment II – survival and growth along an environmental gradient

#### Genotyping and parentage testing

Of the 3687 fish sampled for parental assignment, 3672 were unambiguously identified to family. Hence, 15 individuals were not assigned to family, either due to overlapping composite genotypes between family pairs, or due to genotyping errors. These individual were removed from the data set. A further 19 individuals were post hoc excluded from the growth analysis. Of these 19 individuals, 9 displayed growth malformation and/or extreme condition factor values, while 10 individuals were excluded due to a documented sampling error. Thus, the growth analyses were based upon 3653 individuals in total.

#### Mortality and sampling

**Figure 5:**
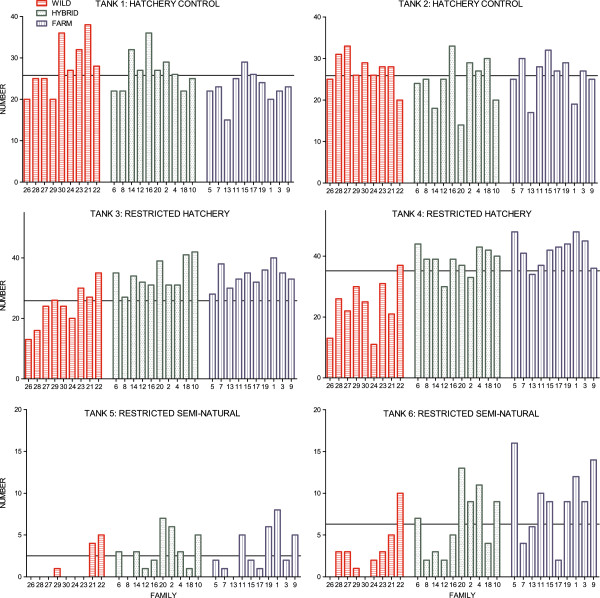
**Family representation in all six tanks, experiment II.** Number of sampled individuals from each farmed, hybrid and wild family, in all six tanks. Mortality was low in the hatchery control treatment, thus at the time of sampling 750 individuals were randomly selected for parental assignment. In the restricted hatchery treatment and the restricted semi-natural treatment all individuals were sampled and identified to family. Families within each group are ranked by their egg size in increasing order, due to the positive relationship between egg size and survival. Lines represent the expected number of individuals/family in each tank, assuming all 29 families were sampled/survived equally. All families were sampled within their expected frequencies in the hatchery control treatment where mortality was low. Mortality were higher in the restricted semi-natural treatment than in the restricted hatchery treatment, and wild salmon displayed significantly higher mortality rates than the hybrid and farmed salmon in both treatments.

Mortality was low in the control treatment throughout the experimental period (Table 1). Variation in mortality between replicates were detected (*x*^*2*^ = 7.56, df = 1, P = 0. 006; Table 1). However, in the subsample from each tank (*n* = 750), salmon of all origins were represented within their expected frequencies (Tank 1: *x*^*2*^ = 5.17, df = 2, P = 0.08; Tank 2: *x*^*2*^ = 1.47, df = 2, P = 0.48; Figure 5), as expected if mortality and sampling were random.

In the restricted hatchery and restricted semi-natural treatments, all surviving individuals were identified to family, thus origin-specific mortality was estimated from the total sample size (adjusted *n* = 5568). For salmon of all origins, survival was lower in the restricted semi-natural treatment, compared to in the restricted hatchery treatment (Tables 4 and 5; Figure 5). In both treatments, survival was lower in the wild salmon, while hybrid and farmed salmon displayed higher and insignificantly different survival (Tables 4 and 5; Figure 5). Thus, no significant genotype-by-environment effect upon survival across the restricted hatchery treatment and the restricted semi-natural treatment was detected (Additional file 6).

**Table 4:** **Weight measurements of *Salmo salar*. L of wild, hybrid and farmed origin, experiment II**

**Physical treatment**	**Origin**	**Tank**	***n***	**W (g)**			**L (cm)**			**K**				**W (g)**			**Weight difference**		**Mortality**
				**Mean**	**Median**	**SD**	**Mean**	**Median**	**SD**	**Mean**	**Median**	**SD**	***n***	**Mean**	**Median**	**SD**	**Absolute (g)**	**%**	**%**
Hatchery control	Farm	1	227	35.94	35.0	7.85	13.98	14.00	0.97	1.29	1.30	0.07	486	35.23	35.0	7.43	NA	NA	NA
		2	259	34.61	35.0	7.01	13.77	13.90	0.96	1.30	1.30	0.08							
	Hybrid	1	266	22.35	23.0	5.85	11.98	12.10	1.06	1.26	1.30	0.07	509	21.43	22.0	5.92	NA	NA	NA
		2	243	20.43	21.0	5.85	11.61	11.80	1.12	1.26	1.30	0.07							
	Wild	1	251	11.31	11.0	5.42	9.50	9.50	1.51	1.21	1.22	0.10	497	10.28	9.0	5.14	NA	NA	NA
		2	246	9.23	8.0	4.63	8.92	8.80	1.36	1.19	1.20	0.12							
Restricted hatchery	Farm	3	338	11.28	9.0	7.47	9.37	9.10	2.02	1.18	1.20	0.11	753	10.77	9.0	7.38	24.46	69.4	18.2
		4	415	10.35	9.0	7.28	9.11	9.10	2.06	1.14	1.20	0.14							
	Hybrid	3	339	6.61	5.0	4.65	7.94	7.50	1.59	1.14	1.20	0.14	725	6.02	4.0	4.35	15.42	71.9	21.2
		4	386	5.50	4.0	4.01	7.54	7.20	1.61	1.07	1.10	0.18							
	Wild	3	212	3.97	3.0	2.66	6.84	6.55	1.17	1.10	1.10	0.16	427	3.77	3.0	2.73	6.51	63.3	48.4
		4	215	3.57	3.0	2.80	6.69	6.50	1.36	1.00	1.10	0.23							
Restricted semi-natural	Farm	5	32	13.59	12.5	6.98	10.00	10.10	1.86	1.23	1.20	0.09	123	11.85	11.0	5.38	23.39	66.4	87.7
		6	91	11.23	11.0	4.58	9.67	9.80	1.40	1.16	1.20	0.10							
	Hybrid	5	31	9.81	8.0	4.18	9.10	9.00	1.35	1.21	1.20	0.08	96	8.34	8.0	3.73	13.09	61.1	90.4
		6	65	7.65	7.0	3.31	8.59	8.60	1.19	1.13	1.10	0.10							
	Wild	5	10	6.50	6.0	2.88	8.05	7.90	1.03	1.17	1.20	0.14	37	5.62	5.0	2.64	4.66	45.3	95.9
		6	27	5.30	5.0	2.52	7.66	7.50	1.06	1.10.	1.10	0.16							

Condition factor (K), length (cm) and weight (g), with standard deviations, and weight difference between the hatchery control treatment and the restricted treatments (absolute and percent).

**Table 5:** Parameter estimates of the selected GLMM best explaining variation in survival in the restricted treatments, experiment II

**Variable**	**Estimate**	**± SE**	***Z***	**P**
Intercept (Farm, Restricted hatchery)	-10.51	2.63	-3.99	0.0001
Hybrid	-0.31	0.18	-1.68	0.09
Wild^1^	-1.11	0.21	-5.37	<0.0001
Restricted semi-natural	-3.79	0.39	-9.72	<0.0001
Egg size^2^	20.31	4.39	4.62	<0.0001

Wald *Z*-statistics of the selected GLMM fitted using Laplace approximation, with tank nested within treatment as a random intercept and 2 x 2 (co) variance matrix allowing for heterogeneity of variance between families across treatments, i.e., random intercept and slope. SE; standard error of estimates. Differences in probability of survival between salmon of farmed, hybrid or wild origin were investigated by re-running the final model while excluding one of the genetic origins at a time, while multiple comparisons were corrected for by the Bonferroni correction, giving an adjusted significance level of 0.017. ^1^ Wild relative to hybrid salmon: estimated value = -0.81 ± 0.22, *Z* = -3.68, P = 0.0002. ^2^ The effect of egg size upon survival was no longer significant in the farmed and hybrid salmon after the Bonferroni correction: estimated value = 15.58 ± 6.66, *Z* = 2.34, P = 0.019.

**Figure 6:**
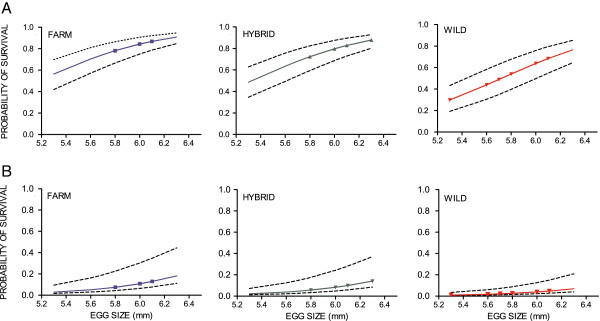
**Probability of survival, experiment II.** Predicted probability of survival in salmon of farmed, hybrid and wild origin against egg size in the **A)** restricted hatchery treatment and **B)** the restricted semi-natural treatment. Egg size in diameter (mm). Symbols illustrate the true egg sizes detected within each origin. Dotted lines illustrate uncertainty caused by the random effect of family and tank. Regression coefficients are given in Table 5.

A positive effect of egg size on survival was detected in the restricted hatchery and restricted semi-natural treatments (Table 5; Figure 6). Thus in general, individuals originating from families with high mean egg size had a higher survival than individuals emerging from families with low mean egg size. When corrected for multiple comparisons, the effect of egg size on survival was no longer significant in the farmed and hybrid salmon (P=0.019, Bonferroni P = 0.017), where survival was highest and variation in egg size smallest (Additional file 7).

Heterogeneity of variance in survival between replicate tanks, and among families across treatments was detected and controlled for in the final GLMM (see Additional file 6; Figure 6).

#### Influence of treatment on body weight at termination

**Figure 7:**
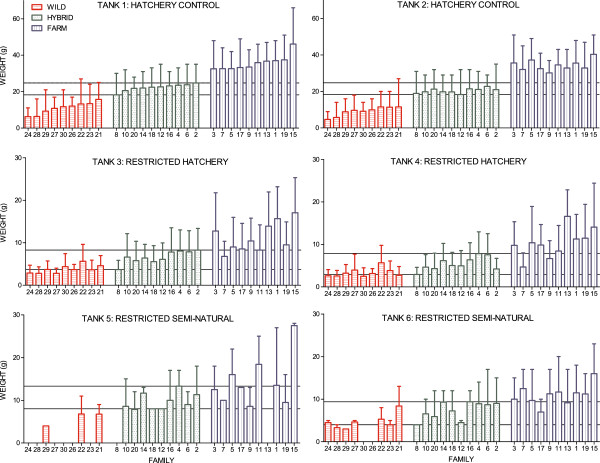
**Mean family weight in all six tanks, experiment II.** Mean family weight (g) of the farmed, hybrid and wild families in all six tanks. Overall fish sizes were higher in the hatchery control treatment, than in the restricted hatchery treatment and the restricted semi-natural treatment. Fish sizes in the restricted hatchery treatment were lower than in the restricted semi-natural treatment. Farmed salmon were significantly larger than the hybrid and wild salmon, in all treatments. There is no overlap in mean family weight of the wild, hybrid and farmed families in the hatchery control treatment, while overlap between families of all origin were detected in the restricted hatchery treatment and the restricted semi-natural treatment. Families are ranked by their mean family weight in tank 1, by increasing order. Lines represent the mean of the smallest and largest hybrid family within each tank. Error bars show the range.

**Figure 8:**
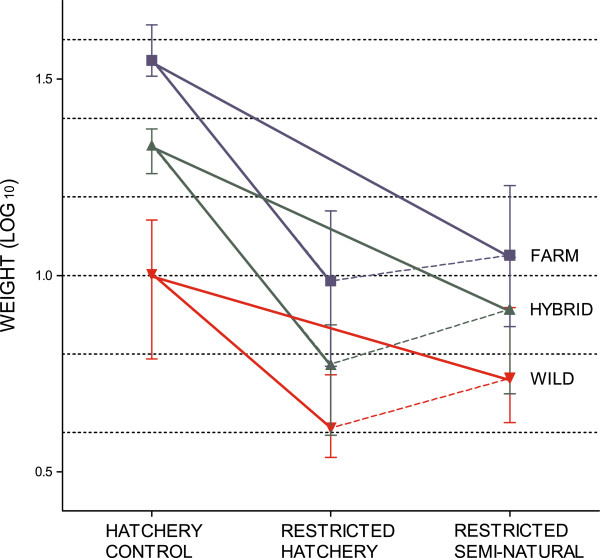
**Growth reaction norms across treatments, experiment II.** Mean weight (log_10_) norm of reaction across all treatments for salmon of farmed, hybrid and wild origin. Replicated tanks are pooled. Error bars show the range of the family means within each experimental group. The elevations of the reaction norms were significantly different between the treatments, as well as between the farmed, hybrid and wild salmon. Wild salmon displayed a significantly flatter negative reaction norm slope between the hatchery control and the restricted hatchery treatments than the hybrid and farmed salmon, which displayed similar and steeper slopes. All groups displayed significantly different reaction norm slopes between the hatchery control and the restricted semi-natural treatment, with farmed salmon displaying the steepest slope, followed by the hybrid salmon. Salmon of all origin displayed similar positive reaction norm slopes between the restricted hatchery treatment and the restricted semi-natural treatment.

Fish size upon termination of the experiment was significantly higher in the hatchery control treatment than in the restricted hatchery treatment and the restricted semi-natural treatment (Tables 3 and 4; Figure 7). Fish size was significantly higher in the restricted semi-natural treatment than in the restricted hatchery treatment, despite the fact that fewer degree days had elapsed in the restricted semi-natural treatment upon termination (Tables 1, 3 and 4; Figure 7). Thus, the general growth reaction norm slope between the hatchery control treatment and both the restricted hatchery treatment and the restricted semi-natural treatment was negative, while the slope between the restricted hatchery treatment and the semi-natural treatment was positive (Figure 8).

Where heterogeneity of variance in body weight at termination between replicate tanks, among families, and among families across treatments was detected, it was controlled for in the final LME model (see Additional file 3). Egg size did not have a significant effect upon body weight at termination, thus the inclusion of egg size in the final LME models did not improve the fit (Additional file 3).

#### Influence of fish origin (farmed/hybrid/wild) on body weight at termination

Body weight of all three experimental groups was significantly different to each other, in all treatments. Farmed salmon were significantly larger than the wild salmon, and hybrids displayed an intermediate body weight that was significantly different to both the farmed and the wild salmon (Tables 3 and 4; Figure 7). Thus the elevation of the growth reaction norms across treatments was significantly different in the three experimental groups (Figure 8).

Significant genotype-by-environment interactions were detected between the hatchery control treatment and both the restricted hatchery treatments and the restricted semi-natural treatment (Table 3). Thus, salmon of farmed, hybrid or wild origin displayed significantly different growth reaction norm slopes. Wild salmon displayed significantly flatter slopes than the hybrid and farmed salmon both between the hatchery control and the restricted hatchery treatments and between the hatchery control and the restricted semi-natural treatments (Figure 8; Additional file 5). The slope between the hatchery control and the restricted hatchery treatments were similar in the hybrid and farmed salmon, while the slope between the hatchery control and the restricted semi-natural treatments were significantly flatter in the hybrid salmon, as compared to the farmed salmon (Figure 8, Additional file 5). Thus, wild salmon, both in the restricted hatchery treatment and the restricted semi-natural treatment displayed a larger relative growth than hybrid and farmed salmon, when compared to growth in the hatchery control treatment (Table 4). Farmed and hybrid salmon displayed similar relative growth in the restricted hatchery treatment, while in the restricted semi-natural treatment hybrid salmon displayed a larger relative growth than the farmed salmon (Table 4). In general, no significant genotype-by-environment interaction was detected between the restricted hatchery treatment and the restricted semi-natural treatment (Additional file 3).

#### Influence of family on body weight at termination

No overlap in mean family weight at termination between the wild, hybrid and farmed salmon were observed in the hatchery control treatment (Figure 7). In the restricted hatchery treatment, overlap in mean family weight between thirteen wild and hybrid families, eleven hybrid and farm families and two wild and farm families were observed (Figure 7). Overlap in mean family weight between families of all three origins was also detected in the restricted semi-natural treatment (Figure 7).

**Figure 9:**
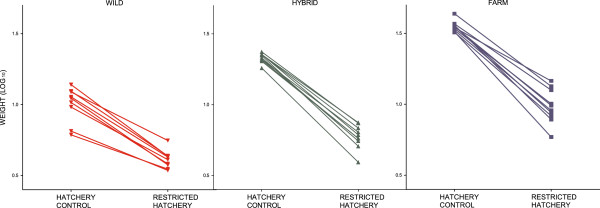
**Family growth reaction norms in the hatchery, experiment II.** Family weight (log_10_) norm of reaction between the hatchery control treatment and the restricted hatchery treatment for salmon of farmed, hybrid and wild origin. Replicated tanks are pooled. A significant correlation between mean family weight in the hatchery control treatment and mean family weight in the restricted hatchery treatment were detected in salmon of all origins.

Salmon of all origin displayed a significant correlation between mean family weight in the hatchery control treatment and mean family weight in the restricted hatchery treatment (Wild: *n* = 36, Pearson r = 0.52, P = 0.001; Hybrid: *n* = 40, Pearson r = 0.52, P = 0.0006; Farm: *n* = 40, Pearson r = 0.47, P = 0.002; Figure 9).

### Heritability of body weight (experiments I and II)

For salmon reared under standard hatchery conditions, a larger portion of the observed phenotypic variation was attributed to genetic variation in the wild salmon, than in the farmed and hybrid salmon (see Figures 4 and 9). Hence a higher heritability estimate *h*^*2*^ of the trait body weight (log_10_) at termination was detected in the wild salmon, than in the farmed salmon, while the lowest heritability estimates were detected in the hybrid salmon (Table 6). Heritability estimates under standard hatchery conditions increased over time (Table 6). Hence, heritability estimates were higher in experiment I, i.e., mixed-strain treatment, than in experiment II, i.e., hatchery control treatment.

**Table 6:** **Heritability of the trait body weight (log**_**10**_**) in salmon of all origins, experiment I and II**

**Experiment**	**Model**	**Social or physical treatment**	**Origin**	***h*** **2**	**HPD interval**	
					**Lower**	**Upper**
I	4.1	Mixed-strain	Farm	0.33	0.16	0.82
	4.2		Hybrid	0.18	0.07	0.65
	4.3		Wild	0.70	0.36	0.92
II	4.4	Hatchery control	Farm	0.16	0.00	0.57
	4.5		Hybrid	0.09	0.00	0.27
	4.6		Wild	0.51	0.10	0.85
	4.7	Restricted hatchery	Farm	0.26	0.02	0.69
	4.8		Hybrid	0.12	0.00	0.44
	4.9		Wild	0.15	0.00	0.43

Heritability *h*^*2*^ of the trait body weight (log_10_) calculated using the animal model, implemented by MCMCglmm. The upper and lower 95% highest posterior density (HPD) intervals represent the credibility intervals of the estimated *h*^*2*^. Models were fitted separately for each experimental group, in all treatments. In addition to the breeding value, *Animal*, the random effect of tank was included in all models as this improved the fit of the MCMCglmm.

In experiment II, heritability estimates of farmed and hybrid salmon increased in the restricted hatchery treatment, while in the wild salmon heritability estimates decreased (Table 6). In addition, phenotypic variation increased in the farmed and hybrid salmon, while decreased in the wild (detectable by visual examination of Figure 9). Thus, larger plasticity between treatments was detected in the farmed and hybrid salmon, as compared to the wild salmon. Decreased phenotypic variation could be expected in the wild salmon if the high mortality were removing the smallest individuals, thus causing an increase in mean body weight of families.

Broad and overlapping credibility intervals, i.e., 95% highest posterior density intervals, were detected in both experiments between all origins. Thus, the trend among the origins, more than the isolated *h*^*2*^ values itself, indicates the difference in genetic variation of the trait body weight at termination among the origins and treatments.

## Discussion

Introgression of farmed escaped salmon represents a major challenge to the genetic integrity of wild populations. It is therefore important to elucidate and quantify the genetic differences between wild and domesticated salmon in order to understand the potential evolutionary consequences of farmed/wild hybridization and introgression in the wild. This study reports the effect of social interaction and inter-strain competition upon growth under standard hatchery conditions in salmon originating from the farmed Mowi strain, the wild Figgjo strain and their F_1_ hybrids. In addition, the norm of reaction for survival and growth along an environmental gradient with differing competitive intensities was investigated. The main results can be summarised as; (i) under standard hatchery conditions the relative difference in growth between farmed and wild salmon was similar when reared separately and together; (ii) growth of surviving farmed, hybrid and wild salmon became more similar as mortality increased along the environmental gradient approaching natural conditions; (iii) under standard hatchery conditions, mean family weight did not overlap between farmed, hybrid and wild families, while overlap between families of all origins were displayed in the restricted treatments.

### Social interaction and inter-strain competition

Mixing salmon of different genetic backgrounds is frequently done in common-garden experiments [16,28,45,46] in order to avoid origin-specific environmental differences, i.e., tank effects. Results of such studies have revealed difference in growth between wild and farmed salmon at ratios as high as 2.9:1 [28] and concern has been raised if these differences are entirely genetically based or influenced by social interaction. This is because origin-specific differences in competitive ability [19] and aggressiveness [16,20,82] cannot be controlled for in common-garden experiments, nor the influence of social dominance and hierarchy [20,83]. In this study, fish in the single-strain treatment were subjected to more movement/handling than the fish in the mixed-strain treatment. Hence temporarily increased stress levels could have affected appetite [84], and as a consequence suppressed growth in salmon of all origins [85]. Although growth across all groups were lower when reared separately, importantly, the relative difference in weight between farmed and wild salmon in this study were similar when reared separately and together. Farmed salmon outgrew wild salmon by a ratio of 5.15:1 and 4.91:1 in the single-strain treatment and mixed-strain treatment, while for hybrid salmon, the adjoining numbers were 1.58:1, and 1.87:1, respectively. These results indicate that the effects of social interaction and inter-strain competition upon growth in studies where salmon of differing genetic backgrounds are reared communally from the eyed-egg stage onwards are not detectably influencing their relative growth rates. Or put simply, the presence of faster-growing farm fish did not impair growth rate of the slower-growing wild fish in this study. Therefore, common-garden experiments appear robust for comparative studies on the farmed/wild interface. It is acknowledged however, that the results of this study could be further validated by conducting growth comparisons of other combinations of strains in order to see if this is generically true.

To our knowledge, this is the first study comparing growth of Atlantic salmon reared in single-strain tanks and mixed-strain tanks from the eye-egg stage, although growth performance of salmon reared in single-family and mixed-family tanks has been compared [86]. In the study by Herbinger and colleagues [86] no relationship between mean family weight in the two rearing environments were detected. In our study, a correlation between mean family weight in the mixed-strain treatment and the single-strain treatment were detected in salmon of all origins. Herbinger and colleagues suggested that environmental differences among replicated tanks had a stronger impact on family growth performance than genetic differences among the families in their study, thus highlighting the benefit of common-garden studies where individuals of all origins are reared together [86]. In contrast to our study, larger weight differences between selected and unselected strains of sea bass *Dicentrarchus labrax* were detected when reared communally, than when reared separately [87]. Although inter-strain competition was likely to contribute to this result, rearing densities in the single-strain tanks were suggested as a stronger regulating factor [87]. This because biomass densities were higher in the selected than the unselected strains when reared separately, potentially resulting in reduced growth and thus smaller differences [87]. Larger growth difference when reared together than separately, have also been documented in progenies of common carp *Cyprinus carpio*[88].

### Survival and growth along an environmental gradient

Surviving wild salmon displayed a flatter growth reaction norm slope between the hatchery control treatment and the restricted hatchery treatment, as compared to the hybrid and farmed salmon. Thus, the relative difference in weight between wild and farmed salmon decreased from a ratio of 3.43:1 to 2.86:1, while the adjoining numbers for hybrid and farmed salmon were insignificantly different between the two treatments at 1.64:1 and 1.79:1, respectively. A larger difference in weight between wild and farmed salmon in the hatchery control treatment could be caused by farmed salmon utilizing feed better than wild salmon [15,89], and thus the *ad libitum* feeding ration benefited the farmed salmon to a larger extent than the wild salmon. However, in the restricted hatchery treatment, mortality was higher in the wild salmon than in the hybrid and farmed salmon, i.e., 48.4, 21.2 and 18.2% respectively. Thus, the flatter slope detected in the wild salmon could also have been caused by the high mortality, if mortality was size-selective towards the slow growing individuals under these conditions. We would then predict size-selective mortality to select against salmon of wild, followed by hybrid origin, while favouring farmed salmon, as mortality increased along our environmental gradient. Consistent with this suggestion, both wild and hybrid salmon displayed significantly flatter growth reaction norm slopes between the hatchery control treatment and the restricted semi-natural treatment, where overall mortality was much higher. Wild salmon displayed a mortality rate of 95.9% in the restricted semi-natural treatment, which is close to mortality rates documented in the wild [14,41]. Although mortality rates were still insignificantly different between hybrid and farmed salmon, i.e., 90.4 and 87.7%, the relative difference in weight between salmon of all origin decreased in the restricted semi-natural treatment where farmed salmon outgrew wild and hybrid salmon by 2.11:1 and 1.42:1, respectively. Thus, growth of surviving farmed, hybrid and wild salmon became more similar as mortality increased along the environmental gradient approaching natural conditions.

The positive relationship observed between egg size and survival were expected, based upon previous studies of Atlantic salmon [41,90] and other salmonids [91,92]. As larger females tend to produce larger eggs [93,94], maternal effects due to differences in size between the wild and farmed parents used in this study are likely to have influenced the low survival rate in the wild salmon where the smallest egg sizes were detected. Egg size has also been documented to be positively related to body size at emergence [91,95], and in the wild even up to >100 days post emergence [90]. As the advantage of emerging from large eggs, upon growth and survival, seems to be stronger under direct competition and suboptimal growth conditions [94,96,97], this could indicate that the strong mortality pressure in the early stages of this experiment, was size-selective towards the smallest individuals, and not origin-specific *per se*.

### Plasticity and genetic variation

Heritability estimates *h*^*2*^ of the trait body weight (log_10_) at termination were higher in the wild, than the hybrid and farmed salmon reared under standard hatchery conditions. Thus a larger portion of the observed phenotypic variation was explained by genetic variation in the wild versus the farmed salmon. Reduced genetic variation for growth in farmed salmon is consistent with the theoretical predictions of domestication [98], and were expected based upon a previous study comparing heritability of this trait in farmed and wild salmon originating from the River Etne, Norway [28].

Unfavourable environmental conditions can increase the estimation of quantitative genetic variation, due to variation being masked under more optimal conditions [99,100]. Consistent with this theory, heritability of the trait body weight of farmed and hybrid salmon increased in the restricted hatchery treatment, as compared to in the hatchery control treatment. However, for wild salmon the heritability estimates decreased. If the high mortality observed in the wild salmon in the restricted hatchery treatment was random, this should have no impact on plasticity or the heritability estimate of the trait body weight. However, if the observed mortality were size-selective as suggested above, e.g., the smallest wild salmon had a lower probability of surviving than the largest wild salmon, plasticity in family growth could appear smaller, resulting in smaller heritability estimates and less genetic variation [101,102]. Thus, the observed decrease in *h*^*2*^ for the wild salmon which showed the highest mortality could indicate that mortality was size-specific in this study.

### Implications

A characteristic feature of domesticated Atlantic salmon is the fact that they outgrow wild salmon extensively under standard hatchery conditions [13,15,16,20,28,37-40]. In this study, farmed salmon outgrew wild salmon at a ratio of 3.43:1 in autumn of their first year and at a ratio of 4.91-5.15:1 in spring the following year (age 1), while the F_1_ hybrid was outgrown by 1.64:1 and 1.58-1.87:1 at the corresponding life stages. In comparison, differences in growth between farmed and wild salmon up to the smolt stage appear to be much smaller in the natural environment. Few studies have investigated differences in growth and survival from hatch in salmon of farmed and wild origin in the wild [14,18,41,42], and in some cases the magnitude of the growth differences are not reported [14,18]. However, in the most comprehensive of these studies which involved measuring family growth and survival in three year classes of salmon planted in the River Guddalselva, Norway, relative differences observed in growth between wild and farmed salmon were 1:1.07, 1:1.25 and 1:1.06 respectively [41]. Further, in another Norwegian study [42], similar small differences in growth were also observed between farmed and wild salmon in the River Imsa. Thus the question arises: why do farmed salmon outgrow wild salmon several-fold under hatchery conditions but only marginally in the natural environment?

This study demonstrates that social interaction and inter-strain competition in densely stocked mixed origin hatchery tanks is not the primary reason as to why farmed salmon in the hatchery outgrow wild salmon in a manner that is not seen in nature. We therefore, tentatively suggest size-selective mortality as a possible underlying factor in reducing differences in growth between wild and farmed salmon in the wild. Size-selective mortality in juvenile teleosts may favour fast growing individuals, as smaller conspecifics have a disadvantage in competition for residence, towards resistance to starvation and parasites, and are more vulnerable for predation [55,103,104], but see [105]. However, reduced anti-predator responses have been documented in farmed and hatchery-reared Atlantic salmon [16,20,21,106] as well as in other domesticated salmonids [56,107,108]. Thus, as farmed salmon is more prone to risk, predation-mortality in the wild could select against the bold fast growing farmed individuals. While negative size-selective mortality then selects against small individuals, positive size-selective mortality selects against large individuals, potentially shifting the populations mean weight towards the middle. Although growth of farmed and wild salmon became more similar as mortality increased along the environmental gradient, this experiment only allowed for negative size-selective competition mortality and not positive size-selective predation mortality. Consequently, further scientific attention on elucidating the selection pressure in the wild is encouraged in order to understand if and to what extent size-selective mortality in the wild could explain why farmed salmon outgrow wild salmon more extensively in the hatchery than in the wild.

## Conclusions

Comparative studies of quantitative traits along the wild/domesticated interface of Atlantic salmon can be used to help understand the potential evolutionary consequences of introgression and hybridization in the wild. This study shows that the large difference in relative growth between wild and farmed salmon detected in the hatchery is similar when reared separately or together. Thus, indicating that social interaction and inter-strain competition are not likely to result in overestimations of genetically based differences in wild and farmed salmon in common-garden experiments. In addition, this study investigated the norm of reaction for survival and growth in Atlantic salmon originating from the farmed Mowi strain, the wild Figgjo strain and their F_1_ hybrids, along an environmental gradient. Restricted feed rations were used to induce nutritional competition in a predator-free-environment, thus allowing for negative size-selective mortality. Based upon the fact that the relative difference in body weight between surviving farmed and wild salmon decreased as mortality increased along the environmental gradient approaching natural conditions, we tentatively suggest a hypothesis that size-selective mortality in the wild could be a possible underlying factor for farmed salmon outgrowing wild salmon less extensively, as compared to in the hatchery. However, further scientific attention on the subject is encouraged.

## Availability of supporting information

The data sets supporting the results of this article are included within the articles additional files (see Additional file 10).

## Competing interests

The authors declare that they have no competing interest.

## Authors’ contributions

MFS and KAG conceived, designed and performed the experiments. MFS, ZZ, FN and KAG analysed the data. MFS, ZZ, FN and KAG wrote the paper. All authors read and approved the final manuscript.

## Supplementary Material

Additional file 1 **Family design.** Family design and parental origin of *Salmo sala*r L. families used in experiment I and II. Click here for file

Additional file 2 **Water temperature November 23, 2010 - March 20, 2012.** Water temperature from family production throughout the study; i) Families are produced and fertilized eggs are incubated in the hatchery; ii) eye-eggs are sorted into experimental groups; iii) eyed-eggs are planted in the semi-natural environment, experiment II (dotted line illustrates the temperature in the semi-natural environment when deviating from the temperature in the hatchery environment); iv) experimental groups are transferred from the hatchery to heated start-feeding tanks, experiment I and II; v) unheated water is supplied from this point and throughout the studies; vi) experiment II is terminated; vii ) experiment I is terminated. Click here for file

Additional file 3 **Model selection LME models.** Model selection on the full LME models describing the effect of treatment, origin and egg size, and all interaction effects, upon body weight (log_10_) at termination, experiment I and II. Click here for file

Additional file 4 **Parameter estimates of the selected LME models, including re-runs.***t*-statistics of the best fitted LME models, using REML estimation, describing the effect of treatment and origin upon body weight (log_10_) at termination, experiment I and II. Click here for file

Additional file 5 **F- statistics of the LME models, including re-runs.** F-statistics of the best fitted LME models, using ML estimation, describing the effect of treatment and origin upon body weight at termination (log_10_), experiment I and II. Click here for file

Additional file 6 **Model selection GLMM.** Model selection on the full GLMM describing the effect of treatment, origin and egg size, and all interaction effects, upon survival in the restricted hatchery treatment and the restricted semi-natural treatment, experiment II. Click here for file

Additional file 7 **Parameter estimates of the selected GLMM, including re-runs.** Wald Z-statistics of the best fit GLMM, fitted using Laplace approximation, describing the effect of treatment, origin and egg size, upon survival in the restricted hatchery treatment and the restricted semi-natural treatment, experiment II. Click here for file

Additional file 8 **Model selection MCMCglmms.** DIC comparisons of the random effect structure of the animal model, implemented by MCMCglmm, experiment I and II. Click here for file

Additional file 9 **Mean family weight in all mixed-strain replicates, experiment I.** Mean family weight (g) of the farmed, hybrid and wild families in all four replicates. There is no overlap in mean family weight of the wild, hybrid and farmed families in any of the four tanks. Families are ranked by their mean family weight in the mixed-strain treatment when replicated tanks are pooled, by increasing order. Lines represent the mean of the smallest and largest hybrid family. Error bars show the range. Click here for file

Additional file 10 **Data set.** Full data set supporting the results of this study, experiment I and II. Click here for file
