# Supplementary figures and images for: Growth reaction norms of domesticated, wild and hybrid Atlantic salmon families in response to differing social and physical environments
Source: BMC Evol Biol. 2013 Oct 28;13:234. doi: 10.1186/1471-2148-13-234 (PMC4231500; doi:10.1186/1471-2148-13-234)

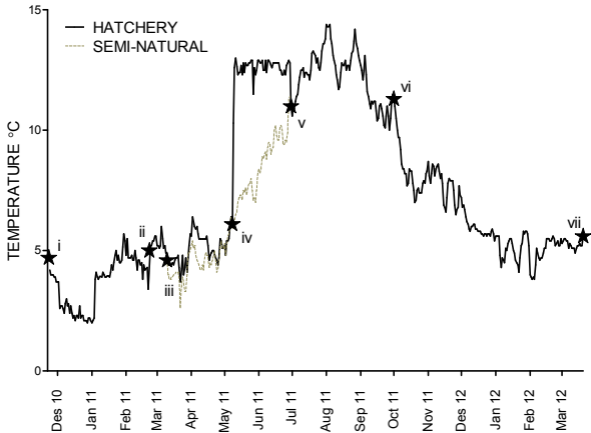

Supplement: Additional file 2 — Water temperature November 23, 2010 - March 20, 2012. Water temperature from family production throughout the study; i) Families are produced and fertilized eggs are incubated in the hatchery; ii) eye-eggs are sorted into experimental groups; iii) eyed-eggs are planted in the semi-natural environment, experiment II (dotted line illustrates the temperature in the semi-natural environment when deviating from the temperature in the hatchery environment); iv) experimental groups are transferred from the hatchery to heated start-feeding tanks, experiment I and II; v) unheated water is supplied from this point and throughout the studies; vi) experiment II is terminated; vii ) experiment I is terminated. [file 1471-2148-13-234-S2.pdf]

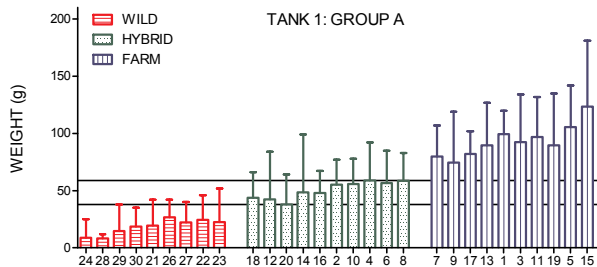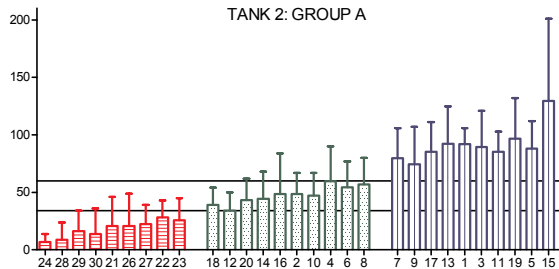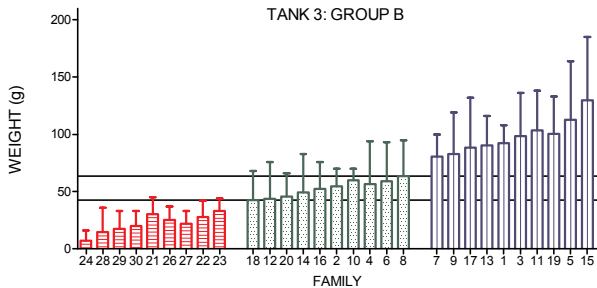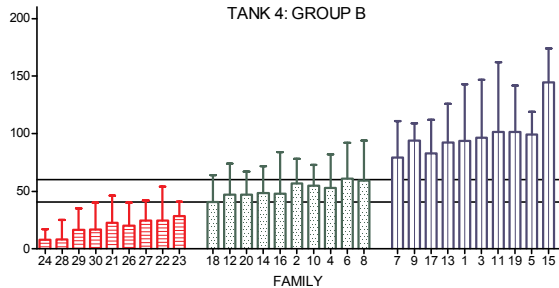

Supplement: Additional file 9 — Mean family weight in all mixed-strain replicates, experiment I. Mean family weight (g) of the farmed, hybrid and wild families in all four replicates. There is no overlap in mean family weight of the wild, hybrid and farmed families in any of the four tanks. Families are ranked by their mean family weight in the mixed-strain treatment when replicated tanks are pooled, by increasing order. Lines represent the mean of the smallest and largest hybrid family. Error bars show the range. [file 1471-2148-13-234-S9.pdf]
